# Guiding principles for the implementation of a standardized psychological autopsy to understand and prevent suicide: a stakeholder analysis

**DOI:** 10.3389/fpsyt.2023.1256229

**Published:** 2023-10-31

**Authors:** Elias Balt, Karlijn Heesen, Arne Popma, Renske Gilissen, Saskia Mérelle, Daan Creemers

**Affiliations:** ^1^Research Department, 113 Suicide Prevention, Amsterdam, Netherlands; ^2^Child and Adolescent Psychiatry & Psychosocial care, Amsterdam University Medical Centre, Amsterdam, Netherlands; ^3^Child and Adolescent Psychiatry, GGZ Oost-Brabant, Boekel, Netherlands

**Keywords:** suicide, prevention, psychological autopsy, implementation, stakeholders

## Abstract

**Background:**

Around 700,000 people die by suicide each year. While the global number of suicides declined over the last decade, the rates remained unchanged in the Netherlands. With this study, we aimed to provide guiding principles for the implementation of a national standardized psychological autopsy to better understand and prevent suicide, by exploring stakeholder perceptions and needs, and barriers to implementation.

**Methods:**

We interviewed 47 representative stakeholders from various fields (e.g., mental healthcare professionals, policy advisors, researchers). A semi structured interview design was used, based on the RE-AIM and Consolidated Framework for Implementation Research (CFIR) theoretical frameworks.

**Results:**

Themes relating to stakeholder perceptions and needs for a standardized psychological autopsy included valorization, accountability, integrability and the needs of the bereaved. Stakeholders believed that participation in a psychological autopsy can help bereaved in their process of grief but noted that evidence to frame the psychological autopsy as postvention is insufficient. The primary focal point should accordingly be to better understand and prevent suicide. Several key limitations of the proposed psychological autopsy approach were detailed, both methodological and implementational.

**Conclusion:**

The stakeholder analysis delineates guiding principles for implementation. Stakeholders believe that a standardized psychological autopsy has merit, provided that key considerations, including valorization and accountability, are integrated in its design. Routine evaluation should be ensured. The findings may guide policy makers and researchers in their endeavors to support a learning, community-based approach for suicide prevention based on a standardized psychological autopsy.

## Background

Suicide is a major global health concern, with approximately 700,000 people who die by suicide annually (1). Approximately 1850 people die by suicide in the Netherlands every year (CBS, 2023). The suicide of a person directly leads to the loss of a life and affects not only the victim’s next-of-kin, but also healthcare professionals, forensic staff, police staff, and bystanders. Indirectly, a suicide creates a ripple effect, causing emotional distress in communities (1), may lead to loss of productivity and economic losses (2, 3), and a deterioration of mental health and increased suicide risk in those who have been affected by the suicide (4–6). Consequently, suicide prevention warrants extensive prevention efforts on a public health level.

The World Health Organization suggests that a multi-level approach is most effective to prevent suicides (1). Our understanding of personal risk factors of suicide and the transition from suicidal thoughts to behaviors is, however, limited, while this would facilitate the development of additional prevention efforts at an earlier stage. Currently, when a suicide occurs in the Netherlands, a coroner determines the cause of death according to the international standards of the ICD-10, and these data are registered. Statistics Netherlands has microdata in which the causes of death can be combined with other characteristics, such as sex, age, household income and educational level. These data are recorded in a national registry. Based on these records, we can determine who died by suicide, but have little insight into *why* they died by suicide. Additionally, when a suicide occurs in a psychiatric hospital or care facility, an evaluation of care is performed. However, this encompasses individuals who received mental healthcare, which in the Netherlands amounts to approximately 40% of all suicide decedents. Ideally, detailed information about a person’s suicide is collected and aggregated on a regional or national level to inform a multi-level, locally integrated approach for prevention (7).

The psychological autopsy is a well-known instrument to obtain information about suicide. Generally, it entails conducting semi-structured interviews with individuals bereaved by the suicide of a loved one and supplementing this information with existing documentation. Performing a psychological autopsy after a suicide provides insight into personal risk factors of a suicide and preceding events (8–12). Moreover, information about suicide trends and conjunctural factors can emerge when routinely executed and aggregated over a longer period. A psychological autopsy into youth suicides has recently been employed in the Netherlands for the first time, exploring its feasibility (13).

International examples of a learning approach for suicide prevention based on a standardized psychological autopsy, like the Suicide Support and Information System (14, 15) and comparable post-mortem monitoring and data collection (16, 17) have yielded in depth insights and tangible recommendations for prevention. However, there has been a paucity of research reporting on challenges to feasibility, standardization procedures and implementation. Several researchers (18–22) have thoroughly reviewed psychological autopsy studies, and state that a protocol for the psychological autopsy is lacking and that methodological issues and limitations require our attention. The authors provide meaningful reflections on striving for scientific rigor and quality norms for the instrument, but do not expand on standardizing procedures for the psychological autopsy method, such as goal setting, stakeholder collaboration, implementation, evaluation, and sustainability of a standardized approach.

Our aim is to establish guiding principles for the implementation of a standardized psychological autopsy. Standardized herein refers to a specific, predetermined set of guiding principles and conditions relating to processes involved in the psychological autopsy, ranging from the interview instrument to data collection and the translation into recommendations for prevention. Implementing a standardized psychological autopsy has not been attempted before in the Netherlands, and conceivably presents considerable challenges. Sustainable implementation requires the collective engagement of stakeholders, which we strive for by involving them in the conceptualization and decision-making processes ahead of implementation planning. We investigated the perceptions and needs of a broad group of stakeholders and explored barriers and facilitators concerning the implementation of a standardized psychological autopsy.

## Methods

### Study design

The stakeholder analysis entailed semi-structured interviews. Stakeholder groups included persons with lived experience, people bereaved by the suicide of next-of-kin, general practitioners, community health care professionals, specialized mental healthcare professionals, policy experts, board professionals of mental health institutions and psychiatric hospitals, suicide prevention and postvention experts, social services professionals, and researchers.

The stakeholders were purposively sampled after receiving consensus of the project team about their conceptual, logistical, financial, or translational involvement with the future implementation, diffusion, and sustainment of the psychological autopsy intervention. Conceptual involvement herein refers to cognitive processes involving the design of the intervention and the implementation strategy. Logistical involvement encompasses activities such as recruitment and data collection. Financial involvement maintains that a stakeholder will be required to invest resources in the intervention. Importantly, the investment of resources may be in the form of the stakeholders’ own time and personnel or may be allocated to finance the activities (conceptual, logistical, translational) of other stakeholders. Translational involvement, lastly, entails a role in the interpretation of data, knowledge exchange, change processes and policy making based on the insights obtained from the intervention.

### Materials

Stakeholders received an information letter about the research, including a preliminary definition of the proposed standardized psychological autopsy.

*“The psychological autopsy is a tool to collect information about suicides. By ‘a standardized approach,’ we refer to implementing the psychological autopsy in accordance with a set of guiding principles and conditions. We aim to define these guiding principles and conditions based on stakeholder perceptions and needs.”*

Interviews were conducted between May 2021 and May 2022. The interview guides for organizational and non-organizational (bereaved people, people with lived experience) stakeholders have been attached with the publication (Tables 1, 2).

The first section of the interview was designed to explore stakeholder perceptions toward implementing the psychological autopsy as the basis of a learning approach for suicide prevention, and to highlight stakeholder needs. The section was guided by the first two questions of this research:

*What are the perceptions of stakeholders relating to the development and implementation of a standardized psychological autopsy approach in the Netherlands?*

*What are the needs of stakeholders relating to the development and implementation of a standardized psychological autopsy approach in the Netherlands?*

Constructs were extracted from two dominant theoretical frameworks in implementation science: the RE-AIM model (23) and the Consolidated Framework for Implementation Research or CFIR (24). These frameworks provided an appropriate theoretical background to investigate implementation constructs, while leaving space to maneuver and explore novel and study-specific concepts.

The RE-AIM model was developed to evaluate the implementation of public health interventions. The model appreciates complex multilevel interventions implemented in community settings, which reflects our intervention goals. Implementation research indicates that the model can similarly be used in pre-implementation planning (26, 27). RE-AIM describes five constructs: reach, efficacy, adoption, implementation, and maintenance. Reach refers to the potential of an intervention to reach the intended target population. Effectiveness relates to the outcomes of the intervention on an individual level, including negative outcomes, and broader outcomes such as quality of life and economic outcomes. Adoption entails the uptake of an intervention in the settings in which the intervention is introduced, and how use of the intervention is facilitated. Implementation refers to the effort to implement the intervention in different contexts, and particularly reflects on how key elements are adhered to and implementation proceeds as intended. Maintenance, lastly, describes the extent to which a program or intervention becomes institutionalized or integrated into routine practice. We operationalized these constructs in relation to stakeholder perceptions and needs. For example, we inquired how stakeholders believe we should inform and recruit bereaved individuals to participate in a psychological autopsy and what would be necessary to achieve this (Reach).

The constructs of *knowledge exchange* and *knowledge translation* were not specifically defined in the RE-AIM model. These constructs are essential to the aim of a standardized psychological autopsy: to learn from the experiences of individuals bereaved by suicide, a complex psychosocial phenomenon, and to aggregate these experiences toward a multi-level approach for prevention through learning networks of community professionals. Therefore, these constructs were adapted from exemplary studies detailing community-based interventions with systematic knowledge exchange and translation to the end of health promotion (28–30).

The second section of the interview aimed to identify barriers and facilitators to the implementation of the psychological autopsy, and was guided by a third research question:

*What perceived barriers and facilitators do stakeholders report to the implementation of a standardized psychological autopsy?*

The CFIR was designed to understand determinants of implementation. Damschroder and colleagues (24) note that the CFIR is commonly employed to evaluate an intervention after implementation, but that the overarching typology of the framework also presents a powerful tool to identify perceived barriers and facilitators in the pre-implementation stages. Open questions were formulated to identify barriers and facilitators from a stakeholder perspective. Follow-up questions detailed the theoretical CFIR domains of [1] intervention characteristics, [2] the inner setting and [3] outer setting, [4] characteristics of individuals and [5] implementation processes. Intervention characteristics are elements of the intervention, including the source, evidence base and relative advantage. The inner setting refers to the setting in which the intervention is implemented, which in this study concerned the organizations of different stakeholders. The outer setting is the broader context in which the inner setting exists, such as a district, state, and incorporates local policy and law. Characteristics of individuals are the needs and roles of individuals involved with the intervention. Implementation processes, lastly, describe all activities and strategies to implement the intervention.

Questions were formulated at a domain level (e.g., *what are barriers to implementing a standardized psychological autopsy, relating to the way [stakeholder organization] functions? These can for example be the organization structure, communicative policies, or the organization culture*. Two considerations informed this approach. Firstly, stakeholders who are less familiar with psychological autopsy may not be able to address barriers relating to narrow constructs. This could elicit responder bias because of the theoretical complexity of constructs. Secondly, this aligns international examples of a pre-implementation assessment of barriers, where barriers are linked to theoretical constructs more specifically in the coding process (27, 31, 32).

### Analysis

Interviews were recorded, transcribed verbatim, and coded using an ATLAS.ti software package (9th edition). Data were analyzed qualitatively in accordance with scientific standards for thematic coding and analysis (25) and employing an adaptation of the Constant Comparative Method (33, 34). The preliminary coding sheet was based on the interview questions and allowed a first round of deductive coding for RE-AIM and CFIR constructs. Through iterative coding cycles, two researchers refined existing codes, and defined codes for emerging themes in an inductive manner. Conflicts were discussed with a third researcher to seek consensus. After coding, the interview data was transposed to a matrix to identify key themes from the interviews through axial comparison.

### Ethical statement

Stakeholders provided written informed consent for the collection, analysis, and publication of the data.

## Results

We conducted 43 interviews with 47 stakeholders. In three interviews, more than one participant attended the appointment. Interviews lasted approximately 1 hour on average. Table 1 presents the study participants. We present key themes relating to [1] the perceptions of stakeholders toward a standardized psychological autopsy and [2] stakeholder needs concerning the implementation. These themes are structured along constructs of the RE-AIM framework. Thereafter, [3] barriers and facilitators to implementation are presented for each of the CFIR domains.

**Table 1:** Stakeholder participants.

Area of expertise	Primary affiliation to study	Organization
Community healthcare and public health	Coroner (3)	Community Health Services
	Project lead suicide prevention (3)	Community Health Services
General healthcare	General practitioner (3)	Independent Practice
	Director (1)	GP psychologists’ interests org.
Specialized healthcare	Psychiatrist (5)	University Medical Center
	Clinical psychologist (2)	Mental Healthcare Facility
	Program manager sp. (1)	Mental Healthcare facility
	Emergency Care Physician (1)	Mental Healthcare Facility
Policy	Policy officer (1)	Ministry of Public health, Welfare and Sports
	Policy advisor (1)	Association of Dutch Municipalities (VNG)
	Ministerial advisor (1)	Advisory Organ for ministry
	Program Director (1)	Inspection for Healthcare and Youth
	Senior inspector (4)	Inspection for Healthcare and Youth
	Project lead sp. (1)	Municipality
	Deputy Mayor (1)	Municipality
Research	Head of research (1)	Road Safety Scientific Research Foundation
	Head of research (1)	Suicide Prevention Organization
	Senior researcher (1)	Suicide Prevention Organization
	Lecturer social psychiatry (1)	University of Applied Sciences
	Researcher (1)	Public health/mental health foundation
	Postvention specialist (1)	Suicide Bereavement and Postvention Team
Social services	Program Officer sp. (1)	Dutch Railway Services
	Psychosocial advisor (1)	Victim Support Netherlands
	Director (1)	Suicide Prevention Organization
	Business Operations Specialist (1)	Dutch National Police
	Psychologist (1)	Dutch National Police
	Project lead sp. (1)	Municipality
	Judicial officer (1)	Tax and customs administration
	Director (1)	Foundation for persons bereaved by suicide
People with lived experience and/or bereaved by suicide	Persons bereaved by suicide (3)	-
	Persons with lived experience (1)	-

Stakeholders were overall supportive of implementing a standardized psychological autopsy in the Netherlands. Themes relating to stakeholder perceptions were *knowledge contribution*, *valorisation*, and *the needs of the bereaved*. Stakeholders highlighted a number of considerations and limitations of the approach, including a saturation of the learning effect. In this study, stakeholder needs refer to what stakeholders reported to value in terms of goals, processes, and outcomes relating to the development and implementation of a standardized psychological autopsy. Central themes concerning stakeholder needs were *representativity*, *accountability*, *learning network*, *clear focal points, uniformity and quality norms*, *integrability* and *evaluation parameters*. Lastly, barriers and facilitators for implementation were discussed. Barriers included *the burden of psychological autopsy for participants and researchers*, *sustainable financing*, and *privacy concerns*. Important facilitators were *clear procedures and a structure for valorisation* and *proper training and guidance for psychological autopsy interviewers*.

### Reach

#### Representativity

Psychological autopsy studies have a risk of selection bias. Stakeholders noted that an adequate representation of completed suicides in the psychological autopsy sample is imperative to achieve generalizable results. They suggest developing a rigorous recruitment and selection procedure, for example by having coroners and general practitioners inform bereaved individuals. This strategy may increase inclusion rates and reduce selection bias compared to other recruitment channels such as social media or organizations for the bereaved, says a senior researcher.

*“[it is] a much more elegant way than a social media post. If every general practitioner would do it, then you can surely include half of the bereaved [in autopsies]. Especially if you want to provide more insight into subgroups, like middle-aged men, you will need bigger numbers [of participants].”*

### Effectiveness

#### Knowledge contribution

Twenty-three stakeholders from various domains (healthcare, social services, research) acknowledged the knowledge contribution of a standardized psychological autopsy. They agreed that the strength of the approach is its potential to determine psychosocial risk factors and precipitating factors for suicide. Specifically, professionals who provide primary care or have a signaling function in society, such as teachers, general practitioners and community care workers believed that the outcomes of psychological autopsies would reinforce their ability to better signal and understand the needs of the help-seeker. The knowledge contribution of a psychological autopsy was particularly emphasized as complementary to existing monitoring and prevention systems, such as population statistics and local suicide monitors. Policy experts and researchers suggested that the psychological autopsy allows for a reconstruction of events and behaviors preceding a suicide and provides insights into risk factors that are currently not yet monitored by default.

Two important limitations were noted by stakeholders relating to knowledge contribution. Firstly, data from psychological autopsies is arguably clouded by the emotions and interpretations of the bereaved, which compromises data validity. The approach is prone to distinct types of bias, such as selection bias, recall bias, social desirability, and confirmation bias. Bereaved individuals may relate specific events or behaviors of the deceased to the suicide because the question implies a relation. Secondly, there were perceived limitations to the learning potential. Three stakeholders questioned if the psychological autopsy would contribute new knowledge at all, and two others stated that saturation will occur after a certain number of interviews, stagnating the initial learning effect.

*“The autopsy may provide all these great insights, and some follow-up care for bereaved is assured, but suppose the approach does not really lead to interventions anymore. All railway crossings have been made safe, by manner of speaking. There will be a point where everything has been done [to prevent suicide], and there will still, always, be people who choose to die.”*

#### Valorisation

Twenty-eight stakeholders stressed that the approach should foremostly lead to improvements in prevention. Ideally, the collected data is translated into comprehensive interventions to prevent suicide. To illustrate this, three stakeholders described a parallel between suicide prevention and the prevention of incidents through safety measures in other field, such as the chemical industry, the flight industry, and the prevention of prenatal death. The core message was consistent: a systematic approach to learn from incidents and consequently improve safety can help better prevent future incidents.

However, psychological autopsies may not always provide compelling evidence to support recommendations, warns a professor of innovation science. Generic or inconsistent knowledge could lead to diffuse recommendations.

*“You suggest that you can signal something that preceded the event [the suicide] which, first of all, is influenced by knowledge you already have [about risk factors] and, secondly, is so diffuse… you cannot go and follow all people who show these signals.”*

Similar remarks were made by a few other stakeholders, suggesting that the gathered insights would be either too subjective or generic to guide suicide prevention efforts.

#### The needs of the bereaved

Stakeholders were concerned with the wellbeing of the bereaved, and frequently balanced the needs of the bereaved against their own. Care for bereaved individuals should be adequate, timely, and diligent, say bereaved individuals, persons with lived experience, researchers, and healthcare professionals. However, stakeholders disputed using the psychological autopsy as a postvention to support bereaved individuals. Some suggested that interviewers may be trained to observe unmet needs of bereaved individuals. Others believed that reflecting on the loss of a loved one could promote a healthy grief process. A mother who lost her child to suicide and participated in a psychological autopsy reflected on the experience and stressed that there is a delicate balance between obtaining scientifically accurate data and meeting needs of the bereaved.

*“It is of course important to leave room for these raw emotions. Because you [as a bereaved person] tell a different story to researchers […]. It helps to tell a story, to have a listening ear. But that does not mean you will get useful information for your research. This is the cord you are balancing on.”*

Most stakeholders felt, however, that a therapeutic effect falls outside the scope of the psychological autopsy. A postvention expert corroborated that the psychological autopsy does not fulfill postvention requirements, and that there is a lack of evidence showing its efficacy to achieve health benefits.

### Adoption

#### Accountability

Ownership of a standardized psychological autopsy should be with one organization according to stakeholder consensus. Preferably this would be a neutral organization with a clear interest in suicide prevention, adequate in-house knowledge and skills, and no commercial motives. However, for the psychological autopsy to contribute to prevention, different stakeholders would need to perceive accountability to utilize the knowledge. A recurring theme was accountability, which refers to a sense of obligation or responsibility, in this case to suicide prevention. While stakeholders had positive perceptions about implementing a standardized autopsy, they warned that if a central organization clearly takes the lead, other stakeholders may not feel compelled to adopt the intervention and adhere to the recommendations for suicide prevention. They stress that accountability must be discussed prior to implementation.

#### Learning network

Ideally, the psychological autopsy evolves into a collaborative network through which information about suicide victims is collected from various sources (school, work, healthcare, and next-of-kin), analyzed, and aggregated by a central organization. Experts from the field then translate the aggregated findings into recommendations. This creates a collaborative network and provides new opportunities for collective prevention efforts. The knowledge should, however, be specific and guided back toward the appropriate stakeholders.

*“A learning network can become a bit vague, when too many parties are involved. […] If there is too much knowledge, diverse knowledge, recommendations become generic. To reach a certain depth [in recommendations for prevention] you need to make knowledge concrete. For example, organize a knowledge exchange session for general practitioners, or in school settings.”*

### Implementation

#### Clear focal points

To facilitate implementation of a standardized psychological autopsy, stakeholders need clarity about what the focal points are. However, there was no consensus about the nature of this focal point. To better understand and prevent suicide was mentioned by 17 stakeholders. A smaller number of stakeholders suggested that the central focus should be to generate knowledge to inform future research, evaluate care, or provide support for the bereaved. Importantly, stakeholders believed it would be inevitable that different stakeholders have their own goals. They warn that missing a clear focal point could lead to deviations in execution, divergence from quality norms, incompatible data, and may hamper adoption of the intervention by stakeholders.

#### Uniformity and quality norms

Twenty-three stakeholders envisioned a uniform approach, explaining how uniformity promotes adherence to quality norms. This ensures that the autopsies produce relevant conclusions and result in feasible recommendations. The data collected must be accessible, specific, and as objective as possible. As an alternative to complete uniformity, a modular approach for the interview was suggested by 18 stakeholders. A list of key indicators for the psychological autopsy is recommended, but other variables may be collected based on the context of the suicide (e.g., inpatient settings) and stakeholder needs. A narrative element, for example, is conceivably imperative to foster empathy and maintain contact with the bereaved respondent. Stakeholders favored mixed-methods data collection, stating that this facilitates both an empathetic and scientifically rigorous approach.

### Maintenance

#### Integrability

Integrability refers to the degree to which the intervention can be integrated with existing initiatives. Nine stakeholders recommended to integrate the standardized psychological autopsy with the evaluation of care that occurs in psychiatric hospitals after a suicide. They suggest this could result in more sustainable implementation. The evaluation after a suicide is currently defensive from the health care provider’s side, and offensive from the side of the bereaved, explains the head of a clinical hospital department. He believes the psychological autopsy could be integrated with the current evaluation to harmonize the perspectives of healthcare professionals and the bereaved, to support a “restorative just culture.” It allows the bereaved to voice their experience and concerns, explains a mother bereaved by suicide.

*“I hope that in psychological autopsy interviews, there will be the opportunity to express a critical view […] reflections that the bereaved have about health care [provided to the deceased]. That those reflections are heard. So that those bereaved by suicide can voice what has happened to them.”*

Crucially, several health care practitioners instead opposed integrating the psychological autopsy into the evaluation of care after a suicide. Firstly, the subjective experience and emotional involvement of the bereaved may conversely lead to false conclusions about quality of care. Secondly, if next of kin will emphasize what they believe to be mistakes made by professionals, this could instead lead to a blame culture, in which health care professionals perceive responsibility for the death of a patient. Lastly, when the autopsy is integrated with the evaluation in specialized care, we would miss crucial knowledge about individuals who have not received specialized care. Insight into these individuals is of foremost importance, as we know little about their suicidal process, and they may have dissimilar needs compared to those in care.

#### Parameters for evaluation

Lastly, eight stakeholders expressed worries about evaluating the intervention for efficacy, which could affect sustainability of the psychological autopsy. It is unlikely that a learning approach involving the psychological autopsy will reduce suicides in the short term. As such, there is a need for different variables to assess its efficacy. However, stakeholders found it difficult to determine appropriate variables to evaluate the intervention. As a result of these limitations, some stakeholders questioned the expediency and feasibility of the intervention.

### Barriers and facilitators for implementation

Stakeholders elucidated barriers to implementation. Subsequently, they discussed facilitating factors. Twenty-five barriers and twenty-one facilitators were described by stakeholders. The barriers and facilitators are indexed in Table 2, along the CFIR constructs. We reflect on the most important barriers and facilitators.

**Table 2:** Barriers and facilitators to the implementation of a standardized psychological autopsy.

CFIR domain	Inner setting					Outer setting					Characteristics of the individual				
Barriers^a^	Resources (time, money) of organizations (9)	Health care professionals fear of being judged and held accountable (7)	Increasing work pressure (3)	Rigid/no open-to-change culture in mental health care (3)		Obstructive privacy laws (17)	Fluctuating political support (5)	Complex decentralized healthcare system with regional differences in priorities (3)	Limited public funds (2)		Perceived risk to wellbeing of bereaved and interviewers (21)	Taboo on talking about suicide (4)	Emotional involvement and feelings of guilt of healthcare professionals and bereaved (4)	Difficult to reach people with migration background (3) and other hard-to-reach groups (2)	Major skill requirements for interviewer/ self-efficacy (2)
Facilitators^a^	Aligning needs of involved stakeholders (7)	A good infrastructure for process and valorisation (6)	Clear ownership of a single organization (4)	Effective collaborative use of funds (4)	Prove effect within setting (pilot) (9)	Early involvement of different stakeholders (5)	Bottom-up approach (2)	Strong legal framework (2)	Create awareness, reduce stigma (2)	Supportive current political climate (2)	A trustworthy ‘brand,’ focused on added value and positive experience (4)	Increase visibility of intervention by campaigns (2)			
CFIR domain	Characteristics of the intervention										Implementation processes				
Barriers^a^	Expensive intervention (15)	Relative advantage limited (9)	Data limitations: difficult to translate to meaningful interventions (7)	Selection bias (6)	Name of the intervention has clinical/forensic implications (5)		Interpretation bias (4)	Validity of qualitative interview data (4)	Evidence base for specific goals is limited (3)	Recall bias of retrospective approach (2)	Learning effect decreases over time (2)	No evaluation parameters and variables (8)	Long term intervention without fixed period (2)		
Facilitators^a^	Proper interviewer training and guidance (7)	Trauma and grief sensitive language: connect on the level of the bereaved (4)	Follow-up after interview (2)	Triangulation of sources (multiple interviews, documentation) (2)	Focus on objective data in aggregation (2)		Multiple regional interview teams (1)					Early engagement of bereaved and bereaved organizations in the process (3)	Change agents for hard-to-reach populations (2)	Support regional comparison (1)	

^a^ The numbers in brackets indicate how many stakeholders reported this barrier or facilitator.

Barriers concerning financial and human resources were frequently noted, entailing the costs of the intervention, the human resources required to execute the many interviews, and the limited funds available to long-term public health initiatives. Some stakeholders believed there were better alternatives, and that the relative advantage was limited considering the investment. Additionally, stakeholders expressed worries about the wellbeing of interviewers and interviewees in psychological autopsies, noting that the experience can be overwhelming. A postvention expert referred to international findings suggesting that intensive suicide-related work such as psychological autopsies may induce suicidal thoughts, which would compromise wellbeing of involved professionals. Obstructive privacy laws were seen as a major barrier in reaching out to bereaved individuals. There is no register in the Netherlands with data about individuals bereaved by suicide. Recruiting through specialized mental health care or social media can induce self-selection bias.

Stakeholders subsequently presented various facilitators of implementation, often framing the as solutions to overcome a formerly described barrier. To meet the needs of interviewers and interviewees, for example, facilitating factors would be proper training and guidance, and follow-up after the interview. To overcome the barrier of sustainable financing, stakeholders suggested to optimize the process, create a firm infrastructure, and ensure effective financial collaboration of stakeholders. The head of a traffic safety research organization explains how the approach can be made more feasible by establishing what would be a representative sample for the autopsies. Based on her work, she suggests creating prototypical scenarios extracted from a combination of risk factors. This allows the formulation of preventive strategies based on smaller samples.

*“We follow a list and eventually come to several factors that played a role in the accident. Then, we group accidents, creating a sort of prototypical scenarios […] following comparable risk factors. For this type of accident, these factors often contribute.”*

Translated to suicide prevention, patterns of risk factors and precipitating factors may be established in an equivalent manner, leading to personalized prevention efforts for identified risk groups.

## Discussion

In the current study we explored the perceptions and needs of stakeholders toward developing and implementing a standardized psychological autopsy in the Netherlands. Stakeholders in our study discussed the themes of knowledge contribution, the needs of the bereaved, accountability, learning networks, clear focal points, uniformity and quality norms, integrability, and evaluation parameters. The most pronounced theme in the interviews was valorisation. Stakeholders postulated that the pinnacle of a learning approach should be to better understand and prevent suicide.

Former research has focused on scientific standards for the psychological autopsy rather than its significance for suicide prevention practices. This is a known challenge in community-based health interventions, where feasibility and stakeholders’ needs must be balanced with scientific rigor (35). One way to improve rigor is the use of a control sample in psychological autopsies. Several exemplary studies have employed case–controls (9, 11, 12, 36, 37). This of course strongly increases the scientific quality and may lead to more valid conclusions and recommendations. However, this was not emphasized as a need by stakeholders in our study. While stakeholders value a uniform approach that is subject to quality norms, they note that the approach should foremostly be practical and lead to recommendations for prevention that may be adopted by the community and specialists.

Additionally, some stakeholders expressed concerns about saturation in learning, worrying that few new insights would be obtained after conducting autopsies for some time. By contrast, most stakeholders agreed that investigating suicides by means of a standardized psychological autopsy elucidates emergent risk factors for suicide and can also help identify and understand trends. The Suicide Support and Information System (14, 15), for example, investigated contemporary societal problems like substance use, mental illness, and unemployment in Ireland, which appreciates suicide in its broader, societal context, and allowed the researchers to provide fitting recommendations for a national agenda for suicide prevention.

The barriers and facilitators delineated by stakeholders present important considerations to plan implementation. Frequently described barriers included characteristics of the intervention and the intervention costs, which may be attributed to the fact that outcomes of a standardized psychological autopsy (i.e., knowledge that may inform recommendations for prevention) may not feel tangible to stakeholders, leading to questions about its feasibility. Nevertheless, stakeholders believed that with an adequate infrastructure for translating the findings into meaningful interventions, valorisation can be achieved, and suicide may be prevented. The World Health Organization’s Live Life guidelines (1) state that multisectoral collaboration with stakeholders, specifically knowledge-sharing, exchange of methodology and lessons learned from previous work, stimulates realistic goal setting, and ensures that recommendations are properly adapted to the local context.

Taken together, the themes addressed by stakeholders produce guiding principles and considerations for the implementation of a standardized psychological autopsy.

1. Define a clear focal point. Focus primarily on valorisation toward suicide prevention.
2. Develop a uniform or modular interview instrument that appreciates the complex nature of suicide. Ensure quality norms. Strive for validity in findings (controls, triangulation).
3. Try to collect objective data but allow bereaved to share their narrative. Maintain anonymity in data and safeguard the privacy of participants. Consider the needs of the bereaved. Proper guidance and support for interviewers must be guaranteed.
4. Generate specific and relevant knowledge. Synthesize with existing data (e.g., population statistics). Formulate feasible recommendations. Support knowledge exchange.
5. Involve stakeholders early. Use the experience and expertise of stakeholders to translate knowledge into prevention efforts. Clearly communicate accountability.
6. Co-create interventions with the field. Account for the needs of the stakeholders and the target population.
7. Strive for tangible outcomes. Define variables for evaluation. Monitor and improve routinely.

Participation in the interview seemed to foster stakeholder engagement, which was expected. Respondents valued the opportunity to share their opinion and concerns at an early stage and said that participating in the interview made them feel part of the conceptualization process. Various experts have stressed the importance and the benefits of early stakeholder involvement in the conceptualization phases and implementation planning (24, 38), which is concurred by our study. In future evaluations, we aim to reflect on stakeholder engagement and involvement.

### Limitations

Several limitations of this research must be considered. Firstly, stakeholders were purposefully selected, imposing a risk of selection bias. The respondents were selected based on a perceived role or interest in suicide prevention, and we aimed to include representatives from different fields. While we did not assess their perceptions toward the psychological autopsy prior to the study, we were obviously familiar with some of their work and interests. To incorporate different perspectives, the research team therefore specifically aimed to balance stakeholders with a known critical view on the psychological autopsy (*n* = 6) and stakeholders who were supportive of the intervention (*n* = 7). Of most stakeholders (*n* = 34), we were not aware of their perceptions. Notably, all involved stakeholders delineated various limitations of a standardized psychological autopsy. Nevertheless, the perception of stakeholders involved in our study may be more positive than in a random sample of stakeholders.

Secondly, we did not include stakeholders looking after the interests of the migrant population. In earlier autopsies in the Netherlands, victims with a migrant background have been underrepresented (13, 39). Historically, researchers have experienced difficulties in including minority populations (40–42). Although several stakeholders discussed procedural strategies to include bereaved of victims with a migrant background, like involving local ambassadors, or community figureheads, we believe that our study would have benefitted from including an expert with knowledge about barriers for migrant populations specifically to participate in suicide research.

## Conclusion

To the best of our knowledge, an exploration of stakeholder perceptions and needs concerning the implementation of a standardized psychological autopsy approach is unprecedented. We provide first directions to the implementation of a standardized psychological autopsy. By using the acclaimed CFIR and RE-AIM theoretical frameworks to guide our inquiry, the identified themes can be more readily translated to different settings. Our findings may guide policy makers and researchers nationally and internationally in their endeavors to implement a learning, community-based approach for suicide prevention based on a standardized psychological autopsy.

## Data availability statement

The raw data supporting the conclusions of this article will be made available by the authors, without undue reservation.

## Ethics statement

Written informed consent was obtained from the individual(s) for the publication of any potentially identifiable images or data included in this article.

## Author contributions

EB: Conceptualization, Data curation, Formal analysis, Methodology, Writing – original draft, Project administration, Writing – review & editing. KH: Conceptualization, Data curation, Formal analysis, Writing – original draft, Writing – review & editing. AP: Conceptualization, Supervision, Writing – review & editing. RG: Conceptualization, Supervision, Writing – review & editing. SM: Conceptualization, Methodology, Supervision, Writing – review & editing. DC: Conceptualization, Methodology, Supervision, Writing – review & editing.
