# Supplementary material for: Guiding principles for the implementation of a standardized psychological autopsy to understand and prevent suicide: a stakeholder analysis
Source: Front Psychiatry. 2023 Oct 31;14:1256229. doi: 10.3389/fpsyt.2023.1256229 (PMC10644706; doi:10.3389/fpsyt.2023.1256229)
Supplement: Supplementary file 1 [file Data_Sheet_1.docx]

Planning the implementation of a standardized psychological autopsy:

A stakeholder analysis

Interview instrument for stakeholders not affiliated with an organization (people with lived experience, bereaved individuals) – final version

**Introduction**

Ask if the participant has read the information letter, and if the participant has any questions relating to the research.

Thank you for your participation in our research. As we described in the information letter, 113 Suicide Prevention is planning the development and implementation of a standardized psychological autopsy. For this interview, we adhere to the following definition of a psychological autopsy.

*“The psychological autopsy is a tool to collect information about suicide. By ‘a standardized approach’, we refer to implementing the psychological autopsy in accordance with a set of guiding principles and conditions. We aim to define these guiding principles and conditions based on stakeholder perceptions and needs.”*

We believe it is essential to involve experts and stakeholders from the field at an early stage in the implementation. Currently, we are in the pre-implementation stages, and we aim to establish the outlines for out implementation planning. The goal of the current study is to identify the perceptions and needs of a broad group of stakeholders, so that we can plan implementation accordingly. That is why we have invited you to participate in this study. Besides establishing stakeholder perceptions and needs, we aim to identify barriers and facilitators to implementation. This allows us to foster our implementation strategy.

By means of a stakeholder analysis, consisting of an interview and a questionnaire, we aim to address these topics. The interview will take approximately 45 minutes of your time. We will start with a couple of questions about your (professional) background. After that, we will ask you about your perceptions and needs relating to a standardized psychological autopsy, how this may align your own [organizational] goals and interest, and we establish what you believe to be important barriers and facilitators to implementation. One day after the interview, we will send you a link to an online questionnaire. This questionnaire proceeds on the topic of barriers and facilitators, inviting you to score the perceived impact of barriers/facilitators. The questionnaire takes approximately 20 minutes to complete.

We would like to emphasize that the psychological autopsy is a flexible research tool and intervention. The exact procedures involved in the execution are not yet detailed. These could be dilemmas such as: who will conduct the interviews in a standardized PA? With which informants? What will be key indicators in the interview? What kind of insights should the interviews yield and how does this contribute to suicide prevention? We invite you to be part of the conceptualization process by asking you to participate in this study. We believe it is crucial that we take your insights, as an important stakeholder, into consideration.

We would like to make a recording of the interview. This is for transcription and analysis purposes only, and your data will be treated as confidential. Only the research team will have access to the recording. Do you agree to us recording the interview, and using your data for the research goals as defined in the information letter?

1. **Background**

*1.1 Bereaved individual/person with lived experience/other non-organizational affiliation*

*1.2 Affiliation to foundation or organization related to suicide prevention (but interview not from organizational perspective)*

*1.3 Would you like to share something about your person experience as a [bereaved individual/person with lived experience]?*

*1.4 Have you ever participated in a psychological autopsy study interview? If yes, what was your experience?*

1. **Perceptions: open**

*The following questions relate to your perceptions about the psychological autopsy as an intervention. We adhere to the definition of the psychological autopsy that was stated at the start of the interview.*

2.1 W*hat are your perceptions about implementing and using a standardized psychological autopsy after suicide?*

*2.2 What, if any, would be the added value of a standardized psychological autopsy after suicides?*

1. **Perceptions and needs: roles**

*3.1 How do you think bereaved individuals would feel about being approached to participate in a psychological autopsy? What thoughts and feelings would be involved?*

*3.2 How do you think bereaved individuals would feel about participating in an interview about the suicide of a loved one?*

*3.3 What do you think is the effect of participating in a psychological autopsy study for bereaved individuals?*

*3.4 What would bereaved individuals need in terms of support and follow-up care when participating in a psychological autopsy study?*

- 1. *Who [among the bereaved individuals] should be informed and recruited for the interviews in the psychological autopsy? [e.g., next of kin, health care professionals]?*
  2. *Who or what organization should inform and recruit bereaved individuals for the psychological autopsy?*
  3. *How can we best reach and inform bereaved individuals about the intervention?
     What is necessary to achieve this?*

3.8 How much time should (minimally) be ensured between the suicide and the interview?

1. **Perceptions and needs: goals and interests**

*4.1 What would, according to you, be the most important goals(s) of a standardized psychological autopsy?*

*4.2 What would be the ideal yields of psychological autopsies? This could, for instance, be insight into numbers and trends, in depth insight into precipitating factors, psychosocial risk factors, themes, insight into at-risk populations, or improved uptake of support by bereaved individuals.*

4.3 How could participating in a psychological autopsy affect feelings of grief in bereaved persons?

4.4 Could the psychological autopsy play a role in the processing of grief for bereaved individuals? What would be needed for this?

*4.5 How should the interview instrument used in autopsies be designed?*

*4.6 What topic [like adverse life events, psychopathology, and so forth] should be included as key indicators in the interview instrument for bereaved individuals in the psychological autopsy?*

*4.6 Do you think that the PA should be conducted according to uniform processes in different settings? Why?*

*4.7 Approximately 40% of suicides in the Netherlands involve persons who received specialized mental healthcare at the time of the death. Around 60% of the victims did not receive specialized care at the time of death. Do you think the instrument for the psychological autopsy must be adapted towards specific populations, or should a uniform approach be employed? Why?*

1. **Knowledge exchange (KE) and knowledge translation (KT)**

*5.1 How should the data obtained from psychological autopsies be stored and managed?*

*5.2 Who should be responsible for the storing and management of the data?*

*5.3 How can we translate the collected knowledge to policy and interventions for suicide prevention?
5.4 How can we best evaluate the effectiveness of the intervention? [Parameters, variables]*

1. **Barriers and facilitators**

*5.1 What do you think are barriers to the implementation of a standardized psychological autopsy in the Netherlands?*

*5.2 What could be barriers specifically for bereaved individuals to participate in psychological autopsy studies?*

*5.3 What could be barriers specifically for bereaved individuals to openly share their intimate stories in psychological autopsy interviews?*

*5.4 What are particularly sensitive subjects to talk about in psychological autopsy interviews?*

*5.5 How can we overcome barriers for bereaved individuals to participate in psychological autopsy interviews?*

*5.6 What would be motivators (facilitating factors) for bereaved individuals to participate in psychological autopsy interviews?*

*5.7 What are facilitating factors to implement a standardized psychological autopsy? These may be facilitators specific to you as a [bereaved person/person with lived experience] or in a broader sense.*

**6. Sustainability**

*6.1 How can we ensure that the psychological autopsy is still conducted after five years?*

6.2 How should the knowledge obtained through psychological autopsies be communicated with bereaved individuals?

*6.3 What are prerequisites to foster the sustainability of a standardized psychological autopsy?*

1. **Perceptions at the end of the interview**
   1. *Did any of your perceptions change throughout this interview? In what way?*

*7.2 Is there anything you would like to add, that has not yet been discussed in the interview?*
